# Profiles of Social Achievement Goals Among Korean High School Students: Associations with Academic Achievement Goals and Emotions

**DOI:** 10.3390/bs16010094

**Published:** 2026-01-09

**Authors:** Boreum Kim

**Affiliations:** Department of Educational Psychology, Ball State University, Indiana, IN 47306, USA; boreum.kim@bsu.edu

**Keywords:** social achievement goals, achievement goals, achievement emotions, social motivation, academic goals, academic motivation

## Abstract

This study explored Korean adolescents’ social achievement goal profiles and their associations with academic achievement goals and achievement emotions. A sample of 1210 high school students completed measures of social achievement goals, 3 × 2 academic achievement goals, and achievement emotions. Latent profile analysis based on three social achievement goals (development, demonstration-approach, and demonstration-avoidance) identified four profiles: Development-Focused Low Social (7%), Development-Focused Moderate Social (49%), High Development and High Avoidance (35%), and Active Socialites (9%). The Development-Focused Low Social profile showed the lowest overall academic achievement goal endorsement, with self-approach goals being most prominent and with lower levels of negative emotions. The Active Socialite group reported the highest academic achievement goals overall, with self- and other-based academic goals most prominent, as well as elevated pride alongside heightened anxiety and shame. Overall, the findings highlight the central role of social motivation in adolescents’ academic goal regulation and emotional experiences within a highly competitive Korean school context.

## 1. Introduction

There is growing recognition that students’ social experiences play a fundamental role in shaping their academic development (Daumiller & Hemi, 2025; Wentzel & Skinner, 2022). Recent studies have demonstrated that social relationship indicators, such as perceived social support, popularity status, and intimacy with peers, are closely linked to students’ academic adjustment (Cui et al., 2025; Schimmelpfennig, 2025; Liem & Fredricks, 2025). Nonetheless, little is known about how social motivation interplays with academic motivation, and studies that focus on the social motivation and its associations with academic outcomes are particularly scarce, whereas the reverse pathway, how academic motivation contributes to social adjustment, has received more attention (e.g., Hemi et al., 2025).

According to Liem and Senko (2022), social goals underlie academic striving and determine students’ academic behaviors. For instance, students may pursue academic success to gain social approval (e.g., pleasing parents) or to attain social recognition (e.g., admiration from peers). The existing findings align with this theoretical assumption, as social achievement goals consistently predict academic outcomes such as attitude toward learning and academic engagement (Liem, 2016; Shim et al., 2025). However, the mechanism of how social achievement goals may form students’ academic experiences have been unclear.

Examining social achievement goals and their role in academic experiences is particularly relevant in the Korean context. Korean schools are characterized by high academic competition, where academic success often carries social meaning. High-achieving students tend to be more accepted among peers, whereas students with lower academic standing are at greater risk of social exclusion or victimization (Abou-ezzeddine et al., 2007; Y. Shin, 2007). In such an environment, students’ social orientations may play a critical role in how they prioritize, pursue, and regulate their academic achievement goals, as well as how they experience emotions in learning contexts. This makes it essential to investigate Korean students’ social achievement goals alongside their academic achievement goals and emotions.

### 1.1. Social Achievement Goals

Social achievement goals refer to individuals’ orientations toward social competence (Ryan & Shim, 2006). This framework includes three types of goals. Social development goals reflect an orientation toward improving one’s social competence; students with these goals focus on learning new skills, growing as social partners, and deepening relationships. These goals involve strengthening social skills, expanding social networks, and cultivating meaningful social relationships. Social demonstration-approach goals involve striving to be positively evaluated by others and to be perceived as socially desirable. Students high in these goals seek positive social feedback to validate their competence. In contrast, social demonstration-avoidance goals reflect efforts to conceal one’s perceived social inadequacies; students who endorse these goals attempt to avoid negative judgments or being seen as socially awkward or ineffective.

A small but growing set of studies has examined social achievement goals in relation to academic outcomes. Findings consistently support the benefits of development goal pursuit, whereas demonstration-based goals show mixed contributions. For example, Liem (2016) found that social development goals were associated with greater academic performance, self-regulated learning, and positive attitudes toward cooperative learning. However, social demonstration-approach goals and demonstration-avoidance goals respectively showed negative and null contributions to self-regulated learning among Indonesian high school students. More recently, Shim et al. (2025) also found that social development goals promote academic engagement (greater involvement and adaptive strategy use and lower maladaptive strategies), whereas both demonstration-approach and demonstration-avoidance goals were detrimental to academic engagement. Despite the current notion on the role of social achievement goals in academic behavior and attitude, little is known about the associations between social achievement goals, academic achievement goals, and achievement emotions.

### 1.2. Academic Achievement Goals

Academic achievement goals are defined as the purposes underlying individuals’ engagement in achievement-related behavior (Maehr, 1989). As a key motivational construct, academic achievement goals contribute to students’ learning experiences, predicting intrinsic motivation, academic achievement, attitudes toward learning, and persistence (Huang, 2011; Rawsthorne & Elliot, 1999; Sideridis & Kaplan, 2011). The achievement goal framework traditionally differentiates mastery goals from performance goals (Ames, 1992; Ames & Archer, 1988; Dweck, 1986). Mastery goals involve striving for task mastery and the development of one’s competence, whereas performance goals involve striving to demonstrate competence, often by outperforming others. Early achievement goal theorists agreed that mastery goals are generally more adaptive than performance goals (Roberts, 1992).

The present study adopts the most recent 3 × 2 academic achievement goal framework (Elliot et al., 2011), which further differentiates mastery goals into two standards of evaluation, task-based and self-based, while performance goals are reconceptualized as other-based goals. Each standard is divided into approach valence, defined as striving to attain a desired outcome, and avoidance valence, defined as striving to avoid an undesired outcome. Thus, the 3 × 2 model consists of six goals: task-approach (doing a task correctly), task-avoidance (avoiding doing a task incorrectly), self-approach (doing better than before), self-avoidance (avoiding doing worse than before), other-approach (doing better than others), and other-avoidance (avoiding doing worse than others). The 3 × 2 framework has been applied across educational settings and cultural contexts (see Lochbaum et al., 2023, for a systematic review). Task-approach and self-approach goals have been documented as adaptive forms of motivation associated with greater engagement, persistence, and deeper cognitive processing (Elliot et al., 2011; Thomas, 2022). In contrast, other-avoidance goals are known to be associated with maladaptive outcomes, including test-irrelevant thinking, tension, and surface-level learning strategies (Thomas, 2022; Üztemur, 2020).

Despite their importance, social achievement goals have received comparatively limited attention in research on academic motivation and emotions. A few studies have integrated both academic and social achievement goals to understand students’ learning experiences (e.g., Bardach et al., 2023; B. Kim et al., 2025; Shim & Finch, 2014), providing valuable insights. However, when both domains are included as profile indicators, it can be difficult to distinguish the unique contribution of social achievement goals, particularly because academic and social goals serve different motivational functions (Urdan & Maehr, 1995; Ryan & Shim, 2006; Wentzel, 1999). Recent work using combined indicators (B. Kim et al., 2025) also suggests that academic achievement goals tend to exhibit greater variability than social achievement goals. Because person-centered solutions can be influenced by the relative variability of included indicators (Marsh et al., 2009; Masyn, 2013), profiles in such models may reduce the visibility of distinct patterns in social motivation. Thus, examining social achievement goals separately allows for a clearer identification of their unique patterns and a more precise understanding of how social motivation relates to students’ academic and emotional experiences.

### 1.3. Achievement Emotions

Achievement emotions refer to emotions directly associated with achievement activities or their outcomes (Pekrun et al., 2011). According to the control value theory (Pekrun, 2006), learners experience different achievement emotions depending on their control-related and value-related appraisals of the achievement situation. Control-related appraisals are perceptions of the controllability of success in achievement activities and outcomes (e.g., when a learner perceives that an achievement activity or outcome is likely to be attained), while value-related appraisals refer to the personal importance and value of achievement activities or outcomes (e.g., when a learner judges an activity or outcome as having high instrumental, usefulness, or attainment values). For instance, enjoyment arises when learners perceive high control and high value in the activity, whereas anger occurs when they perceive low control and low value over the task or its outcome.

Some achievement emotions are inherently social in nature, as they arise in contexts involving social attainment or loss. For example, students may experience enjoyment during class through positive interactions with peers or feel shame when they lose face in front of others. Thus, social achievement goals are likely to be closely linked to such emotions. Pursuing social development goals can enhance the perceived value of classroom activities by providing opportunities to interact with others and to build meaningful relationships with peers and teachers. In addition, pursuing social development goals may increase the likelihood of appraising a class as successful, as students may feel a sense of accomplishment when they believe that the class has helped them develop their social competence. Likewise, pursuing social demonstration-approach goals can increase the perceived value of class activities when they offer opportunities to gain positive attention or recognition from peers and teachers. In contrast, pursuing social demonstration-avoidance goals may lower perceived control and value, as classroom situations can expose one’s social shortcomings, such as speaking awkwardly in front of others.

Existing empirical evidence supports these conceptual assumptions. Social development goals have been found to relate positively to greater enjoyment and other positive emotions, whereas social demonstration-avoidance goals are associated with heightened fear and shame and lower levels of joy (Shim et al., 2013), greater social anxiety (Zhao, 2022), and decreased self-efficacy (Y. R. Kim & Park, 2021). In contrast, social demonstration-approach goals have shown weak or inconsistent associations with most emotions. However, despite these initial findings, research to date has rarely examined social achievement goals within the broader framework of achievement emotions, particularly using person-centered approaches. The present study addresses this important gap by identifying social achievement goal profiles among Korean high school students and examining how these profiles differ in their academic achievement goals and achievement emotions.

Taken together, these considerations highlight the value of profiling social achievement goals independently to enable clearer identification of their specific patterns and their associations with students’ academic achievement goals and achievement emotions. Although E. J. Lee (2018) conducted a latent profile analysis (LPA) focusing on Korean high school students’ social achievement goals, that investigation was limited to the social domain. Therefore, further research is needed to examine how social achievement goal profiles relate to students’ academic motivation and emotional experiences, offering a richer understanding of the interplay between these motivational domains.

### 1.4. Current Study

The present study aimed to identify social achievement goal profiles among South Korean high school students in order to better understand their social motivation and its associations with academic achievement goals and achievement emotions. The following research questions guided the investigation:

*RQ1. What are the social achievement goal profiles of Korean high school students?*

Because the exact number and structure of latent profiles cannot be known a priori in a person-centered approach (Morin et al., 2018; Pastor et al., 2007), expectations were informed by previous findings. Earlier person-centered studies including social goals have generally identified four to six latent groups, including type-specific profiles (e.g., development-oriented or demonstration-oriented), valence-specific profiles (e.g., approach-oriented or avoidance-oriented), and general profiles (e.g., all-low or all-high social goals) (Bardach et al., 2023; B. Kim et al., 2025; Shim & Finch, 2014; E. J. Lee, 2018). Based on this evidence, it was expected to identify four to six latent profile groups reflecting type-specific, valence-specific, and general social goal patterns.

**H1a.**  *Social achievement goal profiles include type-specific groups such as (a) development-focused and (b) demonstration-focused profiles.*

**H1b.**  *Social achievement goal profiles include valence-specific groups such as (c) approach-focused and (d) avoidance-focused profiles.*

**H1c.**  *Social achievement goal profiles include general social goal profiles such as (e) all-low, (f) all-moderate, and (g) all-high endorsement.*

*RQ2. Do social achievement goal profiles differentiate students’ academic achievement goals?*

With respect to RQ2, we anticipated cross-domain links between social and academic achievement goals.

**H2a.**  *Type-specific social goal profiles (development-focused and demonstration-focused) display academic goals that follow a corresponding pattern (task- and self- focused and other-focused).*

**H2b.**  *Valence-specific social goal profiles (approach-focused and avoidance-focused) social achievement goal profiles display academic goals that follow a corresponding pattern (approach-focused and avoidance-focused).*

**H2c.**  *General social achievement goal profiles (all-low, all-moderate, or all-high levels) display academic achievement goals that follow a corresponding pattern (all-low, all-moderate, or all-high).*

*RQ3. Do social achievement goal profiles differentiate students’ achievement emotions?*

For RQ3, the focus was placed on social-related achievement emotions, defined as emotions experienced in classroom contexts that involve the presence or evaluation of others. This category included enjoyment, pride, anxiety, and shame. In contrast, emotions that can arise independently of others, such as hope, anger, hopelessness, and boredom, were examined separately. Although enjoyment may stem from intrinsic interest, it often emerges through interactions with peers and teachers in classroom settings. Similarly, although anxiety can be internally focused, it often has a strong social evaluative component when students anticipate negative judgments. For these reasons, enjoyment and anxiety were conceptualized as social-related achievement emotions in the current study.

**H3a.**  *Type-specific social goal profiles (development-focused and demonstration-focused) display distinguished patterns in social-oriented emotions.*

- Development-focused social achievement goal profiles display greater enjoyment and pride, as well as lower anxiety and shame.
- Demonstration-focused social achievement goal profiles display greater anxiety and shame, as well as lower enjoyment and pride.

**H3b.**  *Valence-specific social goal profiles (approach-focused and avoidance-focused) display distinguished patterns in social-oriented emotions.*

- Approach-focused social achievement goal profiles display greater enjoyment and pride, as well as lower anxiety and shame.
- Avoidance-focused social achievement goal profiles display greater anxiety and shame, as well as lower enjoyment and pride.

**H3c.**  *General social achievement goal profiles at all-low, all-moderate, or all-high levels display achievement emotions that follow a corresponding pattern (all-low, all-moderate, or all-high).*

## 2. Methods

### 2.1. Participants

The sample included 1200 students in Grades 10 and 11 from four high schools, including two coeducational and two all-boys schools (30% female; 54% 10th graders). The city in which the data were collected is one of South Korea’s major metropolitan areas, and its key socioeconomic indicators align closely with national urban norms. For example, the city’s average disposable income (KRW 2.649 million per capita person in 2023; approximately USD 1950–2000 based on 2025 exchange rates) is comparable to the national urban average (KRW 2.554 million in 2023; approximately USD 1880–1930) (Chosun Biz, 2024). School infrastructure indicators also fall within typical national ranges. The student/teacher ratios in high schools (14.7:1) closely match the corresponding national averages of 14.1:1 in 2023 (Korean Statistical Information Service [KOSIS], 2025). Likewise, the region’s high school advancement rate (75.7% in 2023) is nearly identical to the national rate of 74.9% in 2023 (Korean Educational Development Institute, 2024; Ministry of Education [Republic of Korea], 2023). The participating schools were located in a district implementing the high school equalization policy, through which students are randomly assigned to schools via a lottery system. Students were invited to take part in an online survey through posters displayed on campus, and those who participated received a USD 2 convenience store gift card as compensation. Participation was voluntary.

### 2.2. Measures

All questionnaires used in this study had been previously translated and validated for Korean adolescents. Participants responded to all items on a 5-point Likert scale ranging from 1 (Not true of me) to 5 (Very true of me).

#### 2.2.1. Personal Social Achievement Goals

Students’ social achievement goals were measured with the Korean adaptation of the Social Achievement Goal Questionnaire (SAGQ; Ryan & Shim, 2008), validated for Korean adolescents by J. Choi and Park (2018). The instrument contains 18 items organized into three six-item dimensions: social development goals (e.g., “I like it when I learn better ways to get along with friends”), social demonstration-approach goals (e.g., “It is important to me that others think I am popular”), and social demonstration-avoidance goals (e.g., “It is important to me that I don’t embarrass myself around my friends”).

#### 2.2.2. Personal Academic Achievement Goals

Students’ academic achievement goals were assessed using the Korean version of the 3 × 2 Academic Achievement Goal Orientation Scale for Adolescents (Lim, 2013), which adapts and extends items from the original 3 × 2 AGQ (Elliot et al., 2011). The Korean measure includes 24 items (11 from the original scale and 13 newly developed items) organized into six four-item subscales: task-approach (e.g., “I study to know the right answers to the questions on exams”); task-avoidance (e.g., “I study because I don’t want to have wrong answers”); self-approach (e.g., “I study to perform better on exams than I have in the past”); self-avoidance (e.g., “I study because I don’t want to do worse than I should”); other-approach (e.g., “It is important to me to earn a better grade than my peers”); and other-avoidance (e.g., “I study to avoid getting a worse grade than my peers”).

#### 2.2.3. Achievement Emotions

Students’ achievement emotions were measured using selected subscales from the Achievement Emotions Questionnaire (AEQ; Pekrun et al., 2011), translated and validated for Korean middle and high school students by Chun and M. Lee (2017). Eight emotion subscales were included to capture a range of positive and negative academic emotions: enjoyment (4 items; e.g., “I enjoy my class”), hope (4 items; e.g., “I am full of hope for the class”), pride (5 items; e.g., “I am proud of how well I do in class”), anxiety (3 items; e.g., “I worry whether I am able to cope with all my work”), anger (5 items; e.g., “I am so angry that I would like to throw my study task away”), hopelessness (3 items; e.g., “During class, I feel hopeless”), shame (3 items; e.g., “I am ashamed because others understood more of the class than I did”), and boredom (3 items; e.g., “I can’t concentrate because I am so bored”).

### 2.3. Analyses

Data analysis proceeded in three steps. First, confirmatory factor analyses (CFAs) and Cronbach’s alpha coefficients were used to evaluate the factor structure and reliability of all measures. Second, latent profile analysis (LPA) was conducted to identify distinct social achievement goal profiles. Third, the profiles were examined how they were associated with the outcome variables, academic achievement goals and achievement emotions. All analyses were conducted in Mplus 8.4 (Muthén & Muthén, 1998–2021) using the maximum likelihood robust (MLR) estimator. Missing data were minimal (less than 1%) and were handled with Full Information Maximum Likelihood (FIML) under the MLR framework.

#### 2.3.1. Latent Profile Analyses

The LPA solutions, including one to seven social achievement goal profiles, were estimated with the maximum likelihood robust (MLR) estimator. Means and variance of the profile indicators were freely estimated across profiles. To avoid convergence on suboptimal solutions, all LPA models were estimated using 5000 random start values, 1000 iterations, and retaining the 200 best solutions for the final optimization (Hipp & Bauer, 2006). Raw scores were used as LPA indicators to preserve the full variability and original metric of each construct, consistent with recommended practice in mixture modeling (Masyn, 2013) and to avoid the possible distorted results when using estimated values (Marsh et al., 2009, p. 114).

Latent profile models were selected by systematically comparing and testing incremental models to determine the best-fitting solution. Following established practices (Asparouhov & Muthén, 2014; Marsh et al., 2009; Masyn, 2013; Nylund et al., 2007), lower Bayesian Information Criterion (BIC; Schwarz, 1978) and Akaike Information Criterion (AIC; Akaike, 1974) values, significant Lo-Mendell-Rubin likelihood ratio tests (aLMR; Lo et al., 2001) and Bootstrap Likelihood Ratio Tests (BLRTs), and entropy values of 0.80 or higher were preferred. Each model with k profiles was compared to the preceding model with one profile fewer (k-1). Profile sizes were also evaluated to avoid over-extraction; consistent with widely accepted guidelines, a minimum size of 5% of the total sample was considered necessary to ensure interpretive meaningfulness and statistical stability (Nylund-Gibson & A. Y. Choi, 2018). In addition to statistical indicators, model selection incorporated considerations of interpretability, theoretical relevance, and parsimony. For the selected solution, the Student-Newman-Keuls (SNK) ANOVA post hoc multiple-comparison test was applied to examine whether students from different profile groups differed significantly in their levels of social achievement goals.

#### 2.3.2. Prediction of Achievement Goals and Achievement Emotions

Academic achievement goals (i.e., task-approach, task-avoidance, self-approach, self-avoidance, other-approach, and other-avoidance goals) and achievement emotions (i.e., enjoyment, hope, pride, anxiety, anger, hopelessness, shame, and boredom) were included in the LPA framework as distal outcomes. The BCH method (Bakk & Vermunt, 2016; Bolck et al., 2004) was used to estimate the latent means of achievement goals and achievement emotions for each profile and to compare mean differences between profile groups. This approach applies a weighted multiple-group analysis that controls for measurement error in profile assignment and prevents unintended shifts in class membership when external variables are introduced (Nylund-Gibson et al., 2019). The BCH procedure involved two steps: generating and saving BCH weights with covariates (step 1) and then applying these weights in subsequent analyses (step 2), including examinations of associations with covariates and outcome variables. Profile differences in distal outcomes were evaluated using significance tests of pairwise mean comparisons, and Cohen’s *d* values were calculated to facilitate interpretation using the delta method (Chan & Liem, 2023).

## 3. Results

### 3.1. Preliminary Analysis

As part of the preliminary analyses, the normality of the data was assessed, and the psychometric properties of the subscales were evaluated (Table 1). Because LPA is sensitive to extreme values (Spurk et al., 2020), multivariate outliers were screened using Mahalanobis distance (*p* < 0.001). A total of 15 students were removed from the initial sample of 1215 based on this criterion. Confirmatory factor analyses were then conducted separately for each construct. The factor models for academic achievement goals (CFI = 0.96, TLI = 0.95, RMSEA = 0.04, SRMR = 0.04, χ^2^(210) = 585.38, *p* < 0.001), social achievement goals (CFI = 0.96, TLI = 0.95, RMSEA = 0.05, SRMR = 0.07, χ^2^(120) = 473.26, *p* < 0.001), and achievement emotions (CFI = 0.95, TLI = 0.93, RMSEA = 0.04, SRMR = 0.05, χ^2^(435) = 13075.51, *p* < 0.001) demonstrated acceptable model fit based on widely used guidelines (Hu & Bentler, 1999). Although the chi-square statistics were significant, this was expected given the large sample size and does not indicate poor model fit. All factor loadings were statistically significant at *p* < 0.001. Observed skewness ranged from −0.58 to 0.58 and observed kurtosis ranged from −0.54 to 0.45, indicating that responses were normally distributed. Cronbach’s alpha values suggested acceptable to high reliability for most subscales, ranging from 0.70 to 0.91, with two exceptions (i.e., task-approach goals and anxiety) falling slightly below 0.70 (*α* = 0.69 for both).

### 3.2. Model Selection

**Figure A1:**
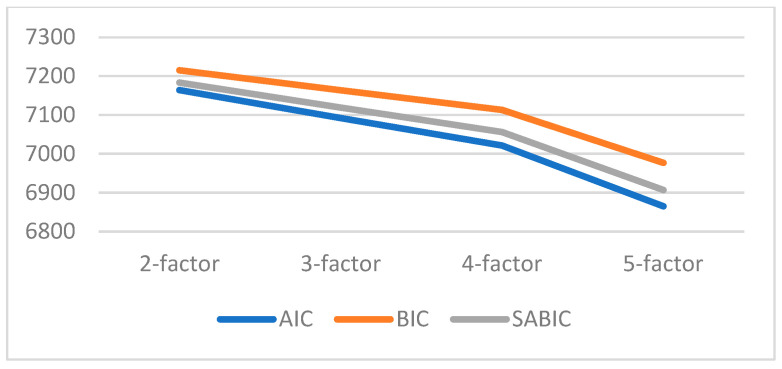
Elbow plot of AIC, BIC, and SABIC across competing factor solutions.

Across the incremental models, AIC, BIC, and SABIC values decreased steadily as additional profiles were added, indicating improved statistical fit (Table 2). The elbow plot (Figure A1) illustrates the improvement across models. Although the five-profile model continued this downward trend and showed slightly better fit indices, it also included a class comprising only 2 percent of the sample. Classes of this size fall below the recommended minimum for stability and meaningful interpretation. In contrast, the four-profile model provided a stronger balance of statistical adequacy and substantive clarity. It demonstrated high entropy (0.82), a significant improvement over the three-profile model based on the LMR test (*p* = 0.02), and class proportions of reasonable size (7% minimum). Taken together, these considerations supported the four-profile model as the most interpretable and defensible solution.

### 3.3. Social Achievement Goal Profiles

**Figure 1:**
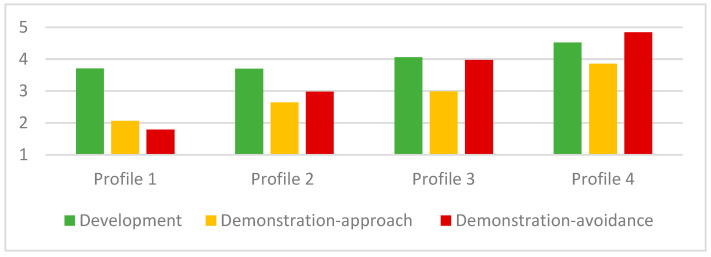
4 Profile solution. Note: Profile 1 = Development-Focused, Disengaged; Profile 2 = Development-Focused, Moderately Disengaged; Profile 3 = High Development and High Avoidance; Profile 4 = Active Socialites.

Table 3 and Figure 1 present the means of social achievement goals for the four-profile model. The first profile group is named the Development-Focused Low Social (N = 92, 7.2%) group. They displayed the lowest social achievement goals, suggesting they are relatively low socially oriented compared to other profiles. However, it is worth noting that their raw score mean for social development goals was moderately high, at 3.71, while their raw score means for social demonstration-approach and -avoidance goals were at extremely low levels, at 2.06 and 1.79, respectively. Despite achieving the lowest social goal engagement in general, their strive for social skills and deepening relationships drives their social orientation.

The second group is the largest profile, named the Development-Focused Moderately Social (N = 583, 49%) group. Their social development goals are not significantly different from the previous profile—at a moderately high level, at 3.70—but their social demonstration goals were higher than the previous profile and were at a moderate level, at 2.64 and 2.98 for demonstration-approach and demonstration-avoidance, respectively. Their social orientation is derived from social development goals, but they have weak orientation on their social competence in relation to others.

The third group is the second largest profile group, High Development and High Avoidance (N = 423, 35%). Their overall social goals are greater than those of the previous two profiles; specifically, their development goals and demonstration-avoidance goals were at a high level, while their demonstration-approach goals were neutral. Although they have greater social development goals, they are concerned with their social incompetence at a similar level.

The last group is Active Socialites (N = 102, 9%). They pursue all social achievement goals at or above a moderately high level. Specifically, their social development and social demonstration-avoidance goals were extremely high, and their desire to show their social competence was moderately high.

### 3.4. Academic Achievement Goals

**Figure 2:**
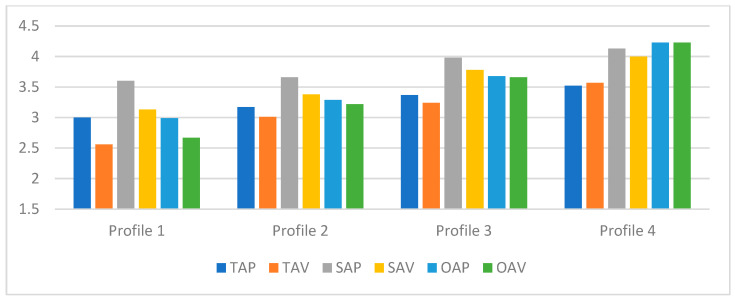
Estimated means of academic achievement goals for each profile. Note: Profile 1 = Development-Focused Low Social; Profile 2 = Development-Focused Moderate Social; Profile 3 = High Development and High Avoidance; Profile 4 = Active Socialites; TAP = task-approach; TAV = task-avoidance; SAP = self-approach; SAV = self-avoidance; OAP = other-approach; OAV = other-avoidance.

Table 4 and Figure 2 present the associations between profile membership and academic achievement goals. The Development-Focused Low Social group (Profile 1) showed relatively low levels across most academic achievement goals. The only exception was self-approach goals, which were moderately high (above 3.5), indicating that students in this profile maintained a personal standard for self-improvement despite otherwise low achievement goal endorsement. For all three standards, approach goals were higher than the corresponding avoidance goals.

The Development-Focused Moderate Social group (Profile 2) displayed task-approach and self-approach goals at levels comparable to Profile 1; however, all other goal types were higher than those of Profile 1 and were above the neutral midpoint (3.01–3.38). Similarly to Profile 1, self-approach goals were the highest within the profile, although differences among the remaining goals were smaller. Approach goals exceeded their corresponding avoidance goals, but the gaps were narrower than those observed in Profile 1.

The High Development and High Avoidance group (Profile 3) demonstrated elevated levels of all academic achievement goals. Consistent with the previous two profiles, self-approach goals were the highest, but this group also reported relatively high levels of self-avoidance, other-approach, and other-avoidance goals (3.66–3.78). Task-approach and self-approach goals exceeded their avoidance counterparts, while other-approach and other-avoidance goals were nearly equivalent.

The Active Socialite group (Profile 4) reported very high levels of self-based and other-based goals (4.00–4.23), along with high task-based goals (3.52–3.57). This pattern suggests strong motivation across both self-fulfilling and competitive dimensions of achievement, rather than a primary focus on task-oriented striving. The differences between approach and avoidance goals were minimal, indicating that a strong desire for self-improvement coexisted with comparable concerns about failing to meet personal standards or underperforming relative to peers.

Pairwise Cohen’s d comparisons demonstrated clear and ordered differences across the four profiles for the most of six goal types. The largest effect sizes were observed in comparisons between the Development-Focused Low Social group (Profile 1) and the Active Socialite group (Profile 4), reflecting the widest contrast in academic achievement goal endorsement.

### 3.5. Achievement Emotions

**Figure 3:**
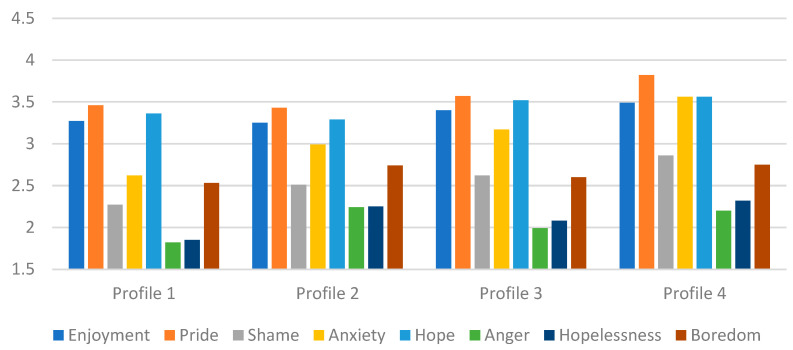
Estimated means of achievement emotions for each profile. Note: Profile 1 = Development-Focused Low Social; Profile 2 = Development-Focused Moderate Social; Profile 3 = High Development and High Avoidance; Profile 4 = Active Socialites.

Table 5 and Figure 3 present the associations between profile membership and achievement emotions. Social achievement goal profiles differed primarily in several social-related achievement emotions, including pride, anxiety, and shame, as well as in two non-social emotions, hopelessness and anger. The Development-Focused Low Social group (Profile 1) showed relatively low levels of pride, shame, anxiety, anger, and hopelessness compared to other groups, whereas their enjoyment, hope, and boredom did not differ significantly from the other profiles. The Development-Focused Moderately Social group (Profile 2) reported lower enjoyment, pride, shame, anxiety, hope, and anger but higher hopelessness and boredom than the High Development and High Avoidance group (Profile 3) or/and the Active Socialite group (Profile 4).

The High Development and High Avoidance group (Profile 3) showed greater enjoyment, pride, shame, anxiety, hope, and anger compared to the Development-Focused Low Social group or/and Development-Focused Moderate Social group, forming an emotional profile characterized by both strong positive emotion and negative emotions compared to the two development-focused groups. The Active Socialite group (Profile 4) demonstrated the highest levels of pride, along with the highest anxiety, suggesting that their strong drive for social standing and interpersonal influence is accompanied by substantial emotional pressure. Their shame, hope, anger, and hopelessness were also higher than in the development-focused profiles.

Across all outcomes, the largest effect sizes were observed for anxiety, which showed the clearest distinctions among the four profiles. In contrast, enjoyment, hope, and boredom displayed minimal or no meaningful differences, indicating that these emotions were less sensitive to variations in social achievement goal patterns.

### 3.6. Supplementary Analyses

Additional analyses were conducted for pride, anxiety, shame, hopelessness, and anger, as these emotions significantly differed across the social achievement goal profiles (see Table A1). Because achievement emotions are also strongly shaped by academic achievement goals, supplementary variable-centered analyses were performed to determine whether the emotional differences observed among profiles could be attributed solely to academic achievement goals or whether social achievement goals contributed uniquely to students’ emotional experiences.

A series of hierarchical regression analyses was conducted for each emotion (pride, anxiety, shame, hopelessness, and anger). Model 1 included the six academic achievement goal dimensions. Model 2 added the three social achievement goal dimensions to examine their incremental predictive value. Model 3 incorporated all interaction terms between academic and social achievement goals (18 interactions), allowing for an examination of whether the joint configuration of academic and social goals explained additional variance.

After accounting for academic goals, social achievement goals continued to demonstrate unique predictive effects in Model 3. Social development goals positively predicted pride (*b* = 0.08, *p* < 0.01) and negatively predicted shame (*b* = −0.09, *p* < 0.01), hopelessness (*b* = −0.07, *p* < 0.01), and anger (*b* = −0.11, *p* < 0.001), indicating that these goals contribute positively to emotional functioning beyond the influence of academic achievement goals.

Several interaction effects emerged. Social development goals were associated with lower shame and anger when combined with high task-approach goals. However, when combined with high task-avoidance goals, social development goals predicted greater shame and anxiety, suggesting that their emotional consequences vary depending on whether students adopt an approach- or avoidance-oriented stance toward task demands. Social development goals also predicted higher anger when paired with high self-approach goals.

Social demonstration-approach goals remained significant predictors in Model 3, positively predicting pride (*b* = 0.08, *p* < 0.01) and anger (*b* = 0.20, *p* < 0.001), indicating a mixed pattern of enhancement and emotional cost. Notably, when social demonstration-approach goals were combined with task-avoidance goals, levels of shame, anger, and hopelessness were lower, suggesting a buffering effect under certain avoidance-oriented academic contexts.

In contrast, social demonstration-avoidance goals showed a detrimental pattern. They negatively predicted pride (*b* = −0.04, *p* < 0.01) and positively predicted anxiety (*b* = 0.10, *p* < 0.001). No meaningful interaction effects were found for social demonstration-avoidance goals, suggesting that their negative emotional influence operates independently of academic achievement goal levels.

Across emotions, Model 3 showed increased explanatory power (R^2^ = 0.24–0.43), indicating that incorporating social achievement goals improved the prediction of achievement emotions beyond academic achievement goals alone.

## 4. Discussion

The present study used a person-centered approach to identify distinct patterns of social achievement goals among Korean high school students and to examine how these patterns relate to academic achievement goals and achievement emotions. Four meaningful profiles emerged: Development-Focused Low Social, Development-Focused Moderate Social, High Development and High Avoidance, and Active Socialites. This study contributes to the literature in several important ways. First, it extends prior achievement goal profile studies (Bardach et al., 2023; B. Kim et al., 2025; Shim & Finch, 2014) by using social achievement goals as the primary indicators for identifying profile groups, thereby highlighting the central role of social motivation in students’ academic adjustment. Second, it clarifies how students’ social motivation is linked to academic motivation by incorporating the 3 × 2 academic achievement goal model, which provides a more differentiated understanding of students’ motivations by distinguishing self-based, task-based, and other-based standards. Third, it applies control-value theory (Pekrun, 2006) to illuminate how different patterns of social motivation are linked to students’ academic emotions. Finally, it advances cross-cultural research on social motivation by focusing on Korean adolescents, a population whose social orientations play a particularly salient role in academic settings yet have received limited empirical attention.

### 4.1. What Are the Social Achievement Goal Profiles of Korean High School Students? (RQ1)

Four distinct social achievement goal profiles were identified. As hypothesized, these included two standard-specific profiles (H1a; Development-Focused Low Social group and Development-Focused Moderate Social group) and one general social profile (H1c; Active Socialite group). However, a purely valence-specific group (H1b) was not observed. Instead, a mixed High Development and High Avoidance profile emerged, which had not been predicted.

#### 4.1.1. Relatively Low Social Motivation but Development-Focused

The current study found two development-focused profiles, the Development-Focused Low Social group (Profile1) and Development-Focused Moderate Social group (Profile 2). The Development-Focused Low Social group reflects students who prefer to remain less visible in class yet value deep and meaningful relationships. Although they show the lowest overall levels of social goals, they report moderate endorsement of social development goals, which are known to support positive school adjustment. Their pattern mirrors the “Undifferentiated Low Profile” identified in E. J. Lee (2018), though in the present study, they represented 7% of the sample (compared to 17% in E. J. Lee’s work). Despite their small size, this group warrants attention because their quiet presence may lead teachers or peers to overlook their developmental strengths and needs.

On the other hand, Development-Focused Moderate Social group, represents students who value deep and meaningful social relationships but show moderate concern about how their social competence is evaluated by peers. Their raw score pattern closely resembles E. J. Lee’s (2018) demonstration-oriented group, which reported means of 3.61 for social development, 3.09 for demonstration-approach, and 2.99 for demonstration-avoidance. The proportion of students in this profile (49%) is also similar to E. J. Lee’s findings (44%). These students do not actively strive for popularity, yet they remain somewhat attentive to how others perceive them, which reflects a development-oriented style that is mindful, but not dominated by, evaluative concerns.

Although these two profiles show relatively low overall social motivation, social development goals were consistently endorsed within each profile. This pattern aligns with Confucian cultural values that emphasize deep interpersonal relationships and group harmony (Zhang et al., 2005). Research in Korean cultural psychology shows that interpersonal norms emphasize sincerity, emotional connectedness, and the cultivation of *jeong* (dʒʌŋ), which refers to deep affective bonds formed through repeated interactions (S. C. Choi & K. Kim, 2006; Yang & Horak, 2019). Korean adolescents may therefore prioritize development goals that help them nurture authentic and trustworthy connections with peers, making social development goals more salient than demonstration-oriented goals.

#### 4.1.2. High Development with High Avoidance

Profile 3, High Development and High Avoidance, was a newly identified profile (35%) that reflects a mixed orientation. Students in this group value deep relationships with development goals but also show high sensitivity to how they appear to others in a concerning perspective with demonstration-avoidance goals. Unlike a purely development-focused or valence-specific group, they combine high development goals with high demonstration-avoidance goals. Their cautious interpersonal style, which involves valuing competence while trying not to stand out, did not appear in E. J. Lee (2018). Instead, Lee identified a High Development and High Demonstration-Approach group (4%). The emergence of a more defensive pattern in the present sample may suggest that students’ social orientations have become more avoidant in recent years due to changes in the social climate, increasing academic pressure, or evolving cultural expectations. Their strong endorsement of avoidance goals may also reflects cultural concerns about maintaining face, preserving interpersonal harmony, and preventing behaviors that may jeopardize group cohesion (Cho et al., 2010; Z. N. Lee, 1999), as well as more recent concerns that Korean adolescents grow up in an environment marked by heightened sensitivity to social evaluation, where even minor deviations from expected behavior can invite criticism and social pressure (H. J. Kim, 2023).

#### 4.1.3. Overall High Social Motivation

Profile 4, Active Socialites, pursues all social goals. This group is comparable to the “Undifferentiated High” goals found in E. J. Lee (2018); however, their demonstration-approach goals are different. Whereas E. J. Lee’s group scored higher demonstration-approach goals than the avoidance counterpart, the present sample showed lower demonstration-approach but higher demonstration-avoidance, suggesting a more defensive approach to social engagement. Also, this group is the only group that actively pursues popularity with demonstration-approach goals above neutral, indicating that only a small proportion of Korean high school students actively seek popularity or social visibility.

Prior research shows that Korean students often avoid overt displays of social dominance or attention-seeking behaviors because these may be viewed as disruptive to group harmony or as violating modesty norms (Hwang, 2012). In addition, the strong academic pressure prevalent in Korean high schools may discourage students from engaging in highly visible social behaviors, as such behaviors may be perceived as inappropriate or as detracting from academic responsibilities (Jarvis et al., 2020; Byun et al., 2012). Given evidence that demonstration-approach and demonstration-avoidance striving may increase aggression or social acceptance (Peets et al., 2022), this profile may face unique socioemotional risks.

### 4.2. Do Social Achievement Goal Profiles Differentiate Students’ Academic Achievement Goals? (RQ2)

Students’ academic achievement goals showed partial alignment with their social achievement goal patterns, which is consistent with prior findings on cross-domain consistency between social and academic goals (Shim & Finch, 2014).

#### 4.2.1. Type-Specific Social Goal Profiles and Academic Achievement Goals (H2a)

This type-congruent hypothesis was partially supported by Development-Focused Low Social group (Profile 1) and Development-Focused Moderate Social group (Profile 2). In the Development-Focused Low Social group, the strongest academic motivation appeared in self-approach goals. This suggests that their desire for self-development in academics parallels their developmental orientation in the social domain. However, their task-based goals remained around the midpoint, indicating that task mastery is not a central academic concern for this development-focused group. This pattern is aligned with the conceptual structure of the 3×2 achievement goal model, in which self-approach goals reflect personal growth and improvement in academic competence and align closely with social development goals reflect personal growth in social competence, whereas task-approach goals emphasize mastering the requirements of the task itself (Elliot et al., 2011).

For Profile 2 (Development-Focused Moderate Social), hypothesis H2a was also partially supported. Their self-approach goals again stood out among all academic goals, suggesting a consistent orientation toward self-development across both domains. Their other-based goals were slightly higher than their task-based goals, which may reflect their moderate endorsement of demonstration-related social goals. In Korean school contexts, where social relationships are closely tied to academic standing (H. Shin, 2020), students in this group may be particularly attentive to academic evaluation as a means of maintaining social status. This profile has consistently emerged as the largest group in prior work (E. J. Lee, 2018) as well as in the present study, highlighting a repeating pattern in which a developmental focus coexists with sensitivity to academic evaluation. Providing support that encourages self-improvement while reducing pressure tied to academic validation may be especially beneficial for these students.

#### 4.2.2. The High Development and High Avoidance Group

This uniquely identified group in the present study, with a High Development and High Avoidance goal, supports the earlier discussion regarding the link between development goals and self-based goals, as well as the connection between demonstration-related goals and other-based goals. Students in this profile showed high levels of both self-based and other-based academic goals. Their strong endorsement of social development goals may be tied to their elevated self-based academic goals, while their heightened social demonstration-avoidance tendencies may contribute to stronger other-based academic goals across valences. Their simultaneous emphasis on self-development in both social and academic domains may promote positive adjustment. However, their pronounced concern about how they are perceived by others in social and academic contexts may also create considerable pressure in their daily school lives. Supporting these students in reducing excessive evaluative concerns while maintaining their developmental focus on personal and social standards may be particularly beneficial.

#### 4.2.3. General Social Achievement Goal Profiles and Academic Achievement Goals (H2c)

For Profile 4 (Active Socialites), the results supported hypothesis H2c. As a general social profile with the highest levels of social achievement goals, this group also exhibited the highest academic achievement goals among all profiles. This pattern is consistent with multiple-goal research indicating that students who endorse several goals tend to demonstrate high motivation across domains (Pastor et al., 2007). Their other-based academic goals were higher than their task-based and self-based goals, suggesting that validating competence in front of peers across both social and academic contexts is especially motivating for this group. Interestingly, this validation tendency appears more passive in the social domain, as reflected in higher avoidance than approach tendencies in social domain, whereas their other-based goals show a more balanced pattern between approach and avoidance. This discrepancy may indicate context-specific pressures to manage social impressions more cautiously while actively striving in academics regardless of both valances.

### 4.3. Do Social Achievement Goal Profiles Differentiate Students’ Achievement Emotions? (RQ3)

#### 4.3.1. Type-Specific Social Goal Profiles and Achievement Emotions (H3a)

The Development-Focused Low Social group (Profile 1) and Development-Focused Moderate Social group (Profile 2) partially supported hypothesis H3a. Students in these profile experienced lower levels of negative emotions, particularly anxiety and shame, compared to the non-development profiles (Profile 3 and Profile 4). Their strong developmental orientation in the social domain may lead them to appraise lower social costs for academic setbacks. Because academic difficulties are unlikely to threaten their primary social pursuit of building deep and meaningful peer relationships, they may feel less anxiety or shame about their academic performance. The supplementary variable-centered analysis supports this claim. Social development goals predicted lower anxiety and shame controlling the effect of academic achievement goals and its interactions with social goals. This interpretation also aligns with prior research demonstrating that social development goals are associated with higher positive affect and lower fear-based emotions at school (Ryan & Shim, 2006; Shim et al., 2013; Wentzel, 2017). However, this group also reported lower pride than the Active Socialite group. This pattern suggests that although their pursuit of social development goals may help buffer negative emotions, it may not be strong enough to enhance positive academic emotions such as pride.

#### 4.3.2. High Development and High Avoidance Group

The High Development and High Avoidance group displayed a mixed emotional pattern. Students in this profile reported elevated pride but also heightened anxiety and shame compared to the two development-focused groups. Pride is a positive appraisal of academic success in the past (Pekrun, 2006). Their strong endorsement of social demonstration-avoidance goals may increase the value they place on their past academic success because excelling academically can serve as a means of protecting or enhancing one’s social reputation. Experiencing academic success could reduce the possibility of social exclusion or relational strain (Cho et al., 2010), concerns that are particularly salient for students with strong demonstration-avoidance tendencies and that may therefore heighten feelings of pride.

However, this heightened concern for avoiding negative evaluation may also intensify fears of losing face, making them more vulnerable to negative emotions. Academic failure may be perceived as jeopardizing peer acceptance or disrupting valued relationships (Z. N. Lee, 1999), which contributes to elevated anxiety and shame. These dynamics align with the interpersonal nature of social achievement goals, particularly within collectivistic and relationship-oriented cultural contexts, where preserving social standing is a central concern (Li, 2016; You & Rud, 2023).

#### 4.3.3. General Social Achievement Goal Profiles and Achievement Emotions (H3c)

The Active Socialite group displayed the highest levels of pride, anxiety, and shame among all profiles. This pattern provides partial support for hypothesis H3c, although several pairwise comparisons were not statistically significant and enjoyment did not differ across groups. Their consistently high endorsement of all three types of social achievement goals suggests that these students are strongly motivated to succeed socially, and that they place substantial value on being positively recognized by peers. At the same time, their elevated social demonstration-avoidance goals indicate a heightened sensitivity to negative evaluation.

This motivational configuration can intensify emotional reactions in both directions. When they succeed, the strong value they place on social recognition may amplify feelings of pride. Pride reflects students’ perceived control over and the value they attach to past success (Pekrun, 2006). For students who strongly endorse social demonstration-approach goals, academic success carries additional social value, such as increased recognition or popularity, which amplifies feelings of pride. In contrast, students with strong demonstration-avoidance goals place greater emphasis on avoiding academic failure and negative evaluation. Because they perceive academic situations as socially risky and feel less control over preventing negative judgments, their pride tends to be dampened regardless of actual performance. Thus, their concern about potential social costs of failure may heighten anxiety and shame, even in situations where failure is only anticipated rather than actual.

In contrast, enjoyment did not differ across profiles. This suggests that positive activating emotions may be less sensitive to social achievement concerns. This may be related to the Korean educational context, where classroom environments do not provide opportunities to establish social relationships. Recent studies note that opportunities for collaboration, discussion, and socially rich learning experiences remain limited in many Korean classrooms (Han, 2025). Such environments may weaken the degree to which social achievement goals shape students’ enjoyment.

### 4.4. Limitations

Although the present study provides meaningful insights into the patterns and roles of social achievement goals on academic achievement goals and achievement emotions, several limitations warrant consideration when interpreting the findings and designing future research. First, the sample was drawn from four high schools within a single metropolitan district operating under the high school equalization policy. Although this policy helps reduce selection effects, the specific geographic and policy context limit the extent to which these findings can be generalized to other regions in Korea or to countries with different competitive climates and school assignment systems.

Second, although enjoyment was conceptualized as a social-related academic emotion, it did not differ significantly across social goal profiles in the present study. This raises the possibility that enjoyment may depend more on classroom-level factors, such as the degree to which teachers structure opportunities for social interaction, than on individual students’ social goal orientations alone. Future research using experience sampling or more context-sensitive designs could clarify how social goal pursuits align with socially grounded emotions like enjoyment.

Third, the analyses did not include covariates. Because social motivation, academic goals, and achievement emotions are influenced by various demographic and contextual factors, the absence of covariate controls limits the ability to determine whether the identified profiles and their associations may differ across demographic groups. Future studies should incorporate gender and age as covariates or examine profile structures separately by subgroup to better capture potential developmental or gender-related differences.

## 5. Conclusions

This study identified four distinct profiles of Korean adolescents’ social achievement goals and demonstrated that these profiles are meaningfully linked to patterns of academic goal orientations and achievement emotions. The profiles ranged from Development-Focused Low Social to Active Socialite, with two intermediate groups differing mainly in the extent to which development-oriented and demonstration-avoidance motives co-occurred. These patterns aligned with academic motivation: type-specific social profiles showed corresponding tendencies in self-, task-, and other-focused academic goals, while the general (all-high) profile displayed uniformly high academic motivation.

Achievement emotions also varied systematically across profiles. The Active Socialite group reported the highest pride but also heightened anxiety and shame, suggesting that strong social striving can enhance motivation while simultaneously increasing emotional strain in competitive school contexts. In contrast, the Development-Focused Low Social group showed the lowest anxiety and shame, reflecting the protective nature of relationally oriented goals when social evaluation concerns are minimal. These trends were further supported by variable-centered analyses, which showed that social development goals predicted lower negative emotions, whereas demonstration-related goals were more closely tied to pride, anxiety, and shame, depending on students’ academic goal orientations.

Overall, the findings underscore that social motivation plays a central role in adolescents’ academic experiences. Incorporating social goal orientations into models of achievement motivation and emotion offers a more complete understanding of students’ school adjustment. Identifying social motivational profiles may help educators provide more targeted support for both academic engagement and emotional well-being in high-pressure school environments.

## Figures and Tables

**Table 1:** Descriptive statistics of social achievement goals, academic achievement goals, and achievement emotions.

	Mean	SD	Skewness	Kurtosis	α
*Social Achievement Goals*					
Development	3.90	0.71	−0.38	0.08	0.89
Demonstration-approach	2.82	0.91	0.23	−0.29	0.91
Demonstration-avoidance	3.39	0.81	−0.14	−0.08	0.87
*Academic Achievement Goals*					
Task-approach	3.26	0.72	−0.17	0.31	0.69
Task-avoidance	3.11	0.74	−0.11	0.31	0.75
Self-approach	3.81	0.73	−0.58	0.45	0.81
Self-avoidance	3.56	0.80	−0.43	0.23	0.80
Other-approach	3.49	0.96	−0.34	−0.41	0.90
Other-avoidance	3.42	0.93	−0.36	−0.25	0.88
*Achievement Emotions*					
Enjoyment	3.33	0.74	−0.02	0.01	0.75
Hope	3.40	0.81	−0.15	−0.21	0.81
Pride	3.51	0.74	−0.12	−0.12	0.78
Anger	2.12	0.78	0.70	0.23	0.79
Anxiety	3.07	0.88	−0.07	−0.31	0.69
Hopelessness	2.16	0.80	0.58	0.12	0.70
Shame	2.56	0.98	0.28	−0.53	0.81
Boredom	2.68	0.86	0.20	−0.18	0.72

**Table 2:** Goodness of fit for models based on different numbers of profile groups.

	Loglikelihood	AIC	BIC	SABIC	Entropy	LMR	Proportion
2-factor	−3571.99	7163.99	7214.89	7183.13	0.58	0.00	72/28
3-factor	−3531.99	7091.97	7163.23	7118.76	0.70	0.00	77/7/16
4-factor	−3492.66	7021.32	7112.94	7055.77	0.82	0.02	7/49/35/9
5-factor	−3410.50	6865.01	6976.99	6907.11	0.88	0.00	25/2/47/9/16

**Table 3:** Means of social achievement goal indicators across profiles.

		Profile 1	Profile 2	Profile 3	Profile 4	S-N-K
SDE	Mean	3.71	3.70	4.06	4.52	1 = 2 < 3 <4
	SD	0.89	0.67	0.62	0.51	
SDAP	Mean	2.06	2.64	2.99	3.86	1 < 2 < 3 < 4
	SD	0.85	0.76	0.84	0.99	
SDAV	Mean	1.79	2.98	3.97	4.84	1 < 2 < 3 < 4
	SD	0.36	0.31	0.28	0.19	

Note: SDE = social development goals; SDAP = social demonstration-approach goals; SDAV = social demonstration-avoidance goals; Profile 1 = Development-Focused Low Social; Profile 2 = Development-Focused Moderate Social; Profile 3 = High Development and High Avoidance; Profile 4 = Active Socialites.

**Table 4:** Estimated means and pairwise standardized differences (Cohen’s d) for academic achievement goals across the four profiles.

	TAP	TAV	SAP	SAV	OAP	OAV
Profile 1	3.00 ^cd^ (0.10)	2.56 ^bcd^ (0.09)	3.60 ^cd^ (0.10)	3.13 ^bcd^ (0.10)	2.99 ^bcd^ (0.13)	2.67 ^bcd^ (0.13)
Profile 2	3.17 ^cd^ (0.03)	3.01 ^acd^ (0.04)	3.66 ^cd^ (0.03)	3.38 ^acd^ (0.03)	3.29 ^acd^ (0.04)	3.22 ^acd^ (0.04)
Profile 3	3.37 ^ab^ (0.04)	3.24 ^abd^ (0.04)	3.98 ^ab^ (0.04)	3.78 ^ab^ (0.04)	3.68 ^abd^ (0.05)	3.66 ^abd^ (0.05)
Profile 4	3.52 ^ab^ (0.10)	3.57 ^abc^ (0.11)	4.13 ^ab^ (0.10))	4.00 ^ab^(0.11)	4.23 ^abc^ (0.12)	4.23 ^abc^ (0.11)
1 _a_ vs. 2	−0.33	−0.88 ***	−0.11	−0.43 *	−0.42 *	−0.82 ***
1 _a_ vs. 3	−0.70 ***	−1.28 ***	−0.72 ***	−1.16 ***	−0.95 ***	−1.44 ***
1 _a_ vs. 4	−0.83 **	−1.55 ***	−0.85 **	−1.26 ***	−1.63 ***	−2.20 ***
2 _a_ vs. 3	−0.44 ***	−0.52 ***	−0.67 ***	−0.83 ***	−0.63 ***	−0.77 ***
2 _a_ vs. 4	−0.60 **	−0.95 ***	−0.81 ***	−0.98 ***	−1.44 ***	−1.68 ***
3 _a_ vs. 4	−0.25	−0.54 *	−0.27	−0.34	−0.83 **	−0.92 ***

Note: Superscript letters indicate a significant difference from profile groups (a = Profile 1, b = Profile 2, c = Profile 3, d = Profile 4) at *p* < 0.05 based on pairwise comparisons. Values in the “1 vs. 2” through “3 vs. 4” rows represent the effect size difference (Cohen’s *d*) between the profiles. Profile 1 = Development-Focused Low Social; Profile 2 = Development-Focused Moderate Social; Profile 3 = High Development and High Avoidance; Profile 4 = Active Socialites; TAP = task-approach; TAV = task-avoidance; SAP = self-approach; SAV = self-avoidance; OAP = other-approach; OAV = other-avoidance. _a_ reference group. * *p* < 0.05, ** *p* < 0.01, and *** *p* < 0.001.

**Table 5:** Estimated means and pairwise standardized differences (Cohen’s d) for achievement emotions across the four profiles.

	Enjoyment	Pride	Shame	Anxiety
Profile 1	3.27 (0.09)	3.46 ^d^ (0.08)	2.27 ^cd^ (0.12)	2.62 ^bcd^ (0.11)
Profile 2	3.25 ^c^ (0.03)	3.43 ^cd^ (0.03)	2.51 ^d^ (0.04)	2.99 ^acd^ (0.04)
Profile 3	3.40 ^b^ (0.04)	3.57 ^bd^ (0.04)	2.62 ^a^ (0.06)	3.17 ^abd^ (0.05)
Profile 4	3.49 (0.12)	3.82 ^abc^ (0.12)	2.86 ^ab^ (0.15)	3.56 ^abc^ (0.13)
1 _a_ vs. 2	−0.04	0.07	−0.35	−0.58 ***
1 _a_ vs. 3	−0.24	−0.21	−0.47 *	−0.83 ***
1 _a_ vs. 4	−0.32	−0.59 *	−0.70 **	−1.23 ***
2 _a_ vs. 3	−0.31 **	−0.30 *	−0.17	−0.32 **
2 _a_ vs. 4	−0.36	−0.67 **	−0.45 *	−0.83 ***
3 _a_ vs. 4	−0.14	−0.42	−0.29	−0.55 *
	Hope	Anger	Hopelessness	Boredom
Profile 1	3.36 (0.10)	1.82 ^bcd^ (0.08)	1.85 ^bcd^ (0.08)	2.53 (0.11)
Profile 2	3.29 ^cd^ (0.04)	1.99 ^ac^ (0.04)	2.25 ^ac^ (0.04)	2.74 ^c^ (0.04)
Profile 3	3.52 ^b^ (0.05)	2.24 ^ab^ (0.04)	2.08 ^ab^ (0.04)	2.60 ^b^ (0.05)
Profile 4	3.56 ^b^ (0.12)	2.20 ^a^ (0.11)	2.32 ^a^ (0.11)	2.75 (0.13)
1 _a_ vs. 2	0.13	−0.84 ***	−0.76 ***	−0.34
1 _a_ vs. 3	−0.27	−0.37	−0.46 *	−0.12
1 _a_ vs. 4	−0.32	−0.64 **	−0.77 ***	−0.3
2 _a_ vs. 3	−0.42	0.48 ***	0.31 **	0.24 *
2 _a_ vs. 4	−0.45 *	0.07	−0.11	−0.01
3 _a_ vs. 4	−0.09	−0.34	−0.38	−0.21

Note: Superscript letters indicate a significant difference from profile groups (a = Profile 1, b = Profile 2, c = Profile 3, d = Profile 4) at *p* < 0.05 based on pairwise comparisons. Values in the “1 vs. 2” through “3 vs. 4” rows represent the effect size difference (Cohen’s *d*) between the profiles. Profile 1 = Development-Focused Low Social; Profile 2 = Development-Focused Moderate Social; Profile 3 = High Development and High Avoidance; Profile 4 = Active Socialites; _a_ reference group. * *p* < 0.05, ** *p* < 0.01, and *** *p* < 0.001.

## Data Availability

The data presented in this study are available on request from the corresponding author.

## Abbreviations

The following abbreviations are used in this manuscript:

SDE	Social Development Goals

## Appendix A

**Table A1:** Hierarchical regression analyses predicting achievement emotions.

		Pride	Anxiety	Shame	Hopelessness	Anger
Model 1	TAP	0.27 ***	−0.25 ***	−0.25 ***	−0.23 ***	−0.12 ***
	TAV	−0.06 ***	0.29 ***	0.29 ***	0.34 ***	0.24 ***
	SAP	0.43	−0.12 **	−0.21 ***	−0.41 ***	−0.37 ***
	SAV	−0.13 ***	0.21 ***	0.14**	0.04 **	−0.05
	OAP	0.33 **	−0.24 ***	−0.28 ***	−0.20 ***	0.00
	OAV	−0.17 ***	0.39 ***	0.41 ***	0.25 ***	0.10
	R^2^	0.40	0.26	0.22	0.24	0.37
Model 2	TAP	0.26 ***	−0.26 ***	−.024 ***	−0.23 ***	−0.12 ***
	TAV	−0.06 ***	0.26 ***	0.28 ***	0.33 ***	0.23 ***
	SAP	0.42	−0.12**	−0.18 ***	−0.36 ***	−0.28 ***
	SAV	−0.13 ***	0.21 ***	0.14 **	0.05 **	−0.04
	OAP	0.31**	−0.23 ***	−0.29 ***	−0.25 ***	−0.09
	OAV	−0.17 ***	0.36 ***	0.40 ***	0.24 ***	0.09
	SDE	0.08 ***	−0.02	−0.09 ***	−0.08**	−0.11 ***
	SDAP	0.08 **	−0.02	−0.01	0.09	0.20 ***
	SDAV	−0.05 **	0.10 ***	0.04*	0.02	−0.04
	R^2^	0.41	0.27	0.22	0.25	0.41
Model 3	TAP	0.25 ***	−0.24 ***	−0.22 ***	−0.20 ***	−0.10 **
	TAV	−0.06 ***	0.26 ***	0.26 ***	0.31 ***	0.22 ***
	SAP	0.43	−0.12**	−0.19 ***	−0.37 ***	−0.29 ***
	SAV	−0.13 ***	0.21 ***	0.14 **	0.08 **	−0.01
	OAP	0.31 **	−0.24 ***	−0.30 ***	−0.26 ***	−0.09
	OAV	−0.17 ***	0.37 ***	0.42 ***	0.24 ***	0.09
	SDE	0.08 ***	−0.01	−0.09**	−0.07 **	−0.11 ***
	SDAP	0.08 **	−0.02	−0.01	0.09	0.20 ***
	SDAV	−0.04 **	0.10 ***	0.05	0.02	−0.04
	SDE XTAP	0.04	−0.08 *	−0.09 *	−0.13 *	−0.11 **
	SDEXTAV	−0.06	0.12 **	0.10 *	0.03 *	−0.02
	SDEXSAP	0.05	0.01	0.03	0.14	0.14 **
	SDEXSAV	0.05	−0.02	−0.01	−0.08	−0.10
	SDEXOAP	−0.09	0.03	0.04	−0.04	−0.02
	SDEXOAV	0.06	−0.04	−0.06	0.04	0.04
	SDAPXTAP	0.04	−0.03	0.01	0.09	0.07
	SDAPXTAV	0.09	−0.13 **	−0.10 *	−0.04 *	0.04
	SDAPXSAP	−0.05 *	0.08	0.03	−0.04	−0.10 *
	SDAPXSAV	−0.04	0.01	−0.01	0.13	0.11 *
	SDAPXOAP	0.00	−0.09	−0.08	−0.04	0.02
	SDAPXOAV	0.00	0.09	0.12*	−0.02*	−0.04
	SDAVXTAP	−0.03	−0.07	−0.05	−0.07	−0.06
	SDAVXTAV	0.01	0.03	0.05	0.01	−0.03
	SDAVXSAP	−0.01 ***	0.04	0.08	0.05	0.10
	SDAVXSAV	0.01 **	−0.06	−0.03	−0.02	−0.07
	SDAVXOAP	−0.01 ***	−0.06	−0.05	−0.02	−0.03
	SDAVXOAV	0.05 *	0.14 **	0.07	−0.02	0.02
	R^2^	0.43	0.29	0.24	0.27	0.43

Note: * *p* < 0.05, ** *p* < 0.01, and *** *p* < 0.001.
